# Supplementary material for: The interactome of intact mitochondria by cross-linking mass spectrometry provides evidence for coexisting respiratory supercomplexes
Source: Mol Cell Proteomics. 2017 Dec 8;17(2):216–32. doi: 10.1074/mcp.RA117.000470 (PMC5795388; doi:10.1074/mcp.RA117.000470)
Supplement: Supplemental Data [file supp_RA117.000470_133922_0_supp_23978_fzffwf.zip › spectra_annotation/mito_DR_spectra_annotation/166-1-6-1-3-1.pdf]

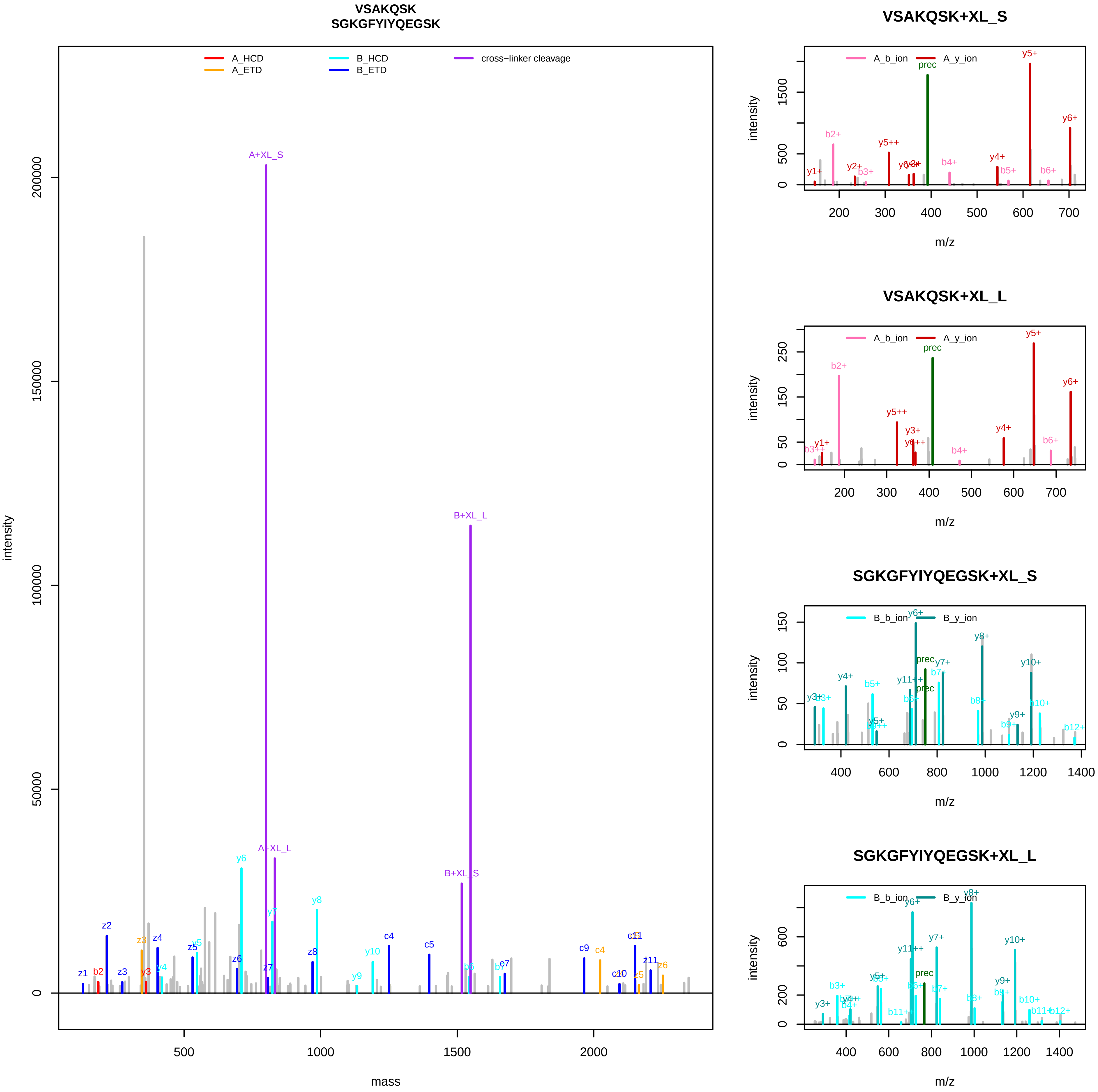

VSAKQSK
SGKGFYIYQEGSK
A_HCD
A_ETD
B_HCD
B_ETD
cross−linker cleavage
intensity
mass
0
50000
100000
150000
200000
500
1000
1500
2000
A+XL_S
B+XL_L
A+XL_L
B+XL_S
z1
b2
z2
z3
y3
z4
z5
y5
y4
y6
z6
z7
y7
y8
z8
y9
y10
c4
c5
b6
b7
c7
c9
c10
c11
z11
z5
z6
VSAKQSK+XL_S
A_b_ion
A_y_ion
prec
y5+
y6+
b2+
y5++
y2+
b3+
y6++
y3+
b4+
y4+
b5+
b6+
y1+
m/z
VSAKQSK+XL_L
b3++
SGKGFYIYQEGSK+XL_S
B_b_ion
B_y_ion
y7+
y8+
y10+
y11++
b7+
b8+
b9+
b10+
b12+
y9+
SGKGFYIYQEGSK+XL_L
b11+
b11++
b12+
